# Rehabilitation of an athelete with Kienbock’s disease

**DOI:** 10.1186/1753-6561-9-S3-A106

**Published:** 2015-05-19

**Authors:** Janis Yeo Siew Ting

**Affiliations:** 1 Department of Occupational Therapy, Tan Tock Seng Hospital, 308433, Singapore

## Introduction

Kienböck’s disease, a vascular necrosis of the lunate, has remained controversial in its basic etiology, natural history and therefore treatment.

## Purpose

This case report describes the hand therapy process of improving the range and strength of a 17-year-old female gymnast to return her to competitive gymnastic training and the competition arena.

## Methods

17-year-old female gymnast, left hand dominant, started hand therapy 8 weeks after a vascularised medial femoral trochlea osteochrondral flap reconstruction to her right wrist.

Initial status, 8 weeks post-operation: AROM fingers full; AROM wrist extension 20°; AROM wrist flexion 15°; AROM wrist radial deviation 8°; AROM wrist ulnar deviation 8°; AROM pronation 70°; AROM supination 90°; *Quick*DASH disability / symptom score 47.7; *Quick*DASH sports module score 100. Patient underwent bi-weekly therapy sessions for first 4 weeks focusing on dart-throwers motion, A/PROM, therapeutic exercise and modalities. This is followed by weekly sessions focusing on strengthening and simulation of gymnastic training.

## Results

16 weeks post-operation: AROM fingers full; AROM / PROM wrist extension 45° / 50°; AROM / PROM wrist flexion 35° / 50°; AROM wrist radial deviation 15°; AROM wrist ulnar deviation 25°; AROM pronation 80°; AROM supination 90°; right grip / left grip strength 13.3kg / 21.7kg; *Quick*DASH disability / symptom score 6.8; *Quick*DASH sports module score 50.

## Conclusion

During the process of rehabilitation, while range of motion and strength remains the tenet of hand therapy, emphasis was also placed on unloading and maintaining the blood supply to the wrist, specifically to the lunate.

